# Magnesium Regulates Endothelial Barrier Functions through TRPM7, MagT1, and S1P1

**DOI:** 10.1002/advs.201901166

**Published:** 2019-07-30

**Authors:** Donghui Zhu, Jing You, Nan Zhao, Huaxi Xu

**Affiliations:** ^1^ Department of Biomedical Engineering Institute for Engineering‐Driven Medicine College of Engineering and Applied Sciences Renaissance School of Medicine Stony Brook University Stony Brook NY 11794 USA; ^2^ Department of Biomedical Engineering University of North Texas Denton TX 76207 USA; ^3^ Department of Biomedical Engineering Pennsylvania State University State College PA 16802 USA; ^4^ Sanford Burnham Prebys Medical Discovery Institute La Jolla CA 92037 USA

**Keywords:** endothelial dysfunction, Mg‐deficiency, vascular biology, vascular endothelium, vascular permeability

## Abstract

Mg^2+^‐deficiency is linked to hypertension, Alzheimer's disease, stroke, migraine headaches, cardiovascular diseases, and diabetes, etc., but its exact role in these pathophysiological conditions remains elusive. Mg^2+^ can regulate vascular functions, yet the mechanistic insight remains ill‐defined. Data show that extracellular Mg^2+^ enters endothelium mainly through the TRPM7 channel and MagT1 transporter. Mg^2+^ can act as an antagonist to reduce Ca^2+^ signaling in endothelium. Mg^2+^ also reduces the intracellular reactive oxygen species (ROS) level and inflammation. In addition, Mg^2+^‐signaling increases endothelial survival and growth, adhesion, and migration. Endothelial barrier integrity is significantly enhanced with Mg^2+^‐treatment through S1P1‐Rac1 pathways and barrier‐stabilizing mediators including cAMP, FGF1/2, and eNOS. Mg^2+^ also promotes cytoskeletal reorganization and junction proteins to tighten up the barrier. Moreover, Mg^2+^‐deficiency enhances endothelial barrier permeability in mice, and Mg^2+^‐treatment rescues histamine‐induced transient vessel hyper‐permeability in vivo. In summary, Mg^2+^‐deficiency can cause deleterious effects in endothelium integrity, and Mg^2+^‐treatment may be effective in the prevention or treatment of vascular dysfunction.

## 1 Introduction

As the second most abundant intracellular divalent cation, magnesium (Mg^2+^) is an indispensable element for human health. It participates in >500 metabolic reactions as a cofactor, including DNA and RNA formation, protein composite, cellular energy supply, reproduction, and mitochondrial membrane stabilization.^1^ It is also involved in preserving normal functions of nerves and muscles, neuromuscular conduction, cardiac excitability, muscular contraction, blood pressure, vasomotor tone, bone function, and glucose related metabolism.[qv: 1a,2] Therefore, Mg^2+^‐deficiency has been linked to many disorders, such as Alzheimer's disease, migraine headaches, cerebrovascular abnormalities, diabetes, hypertension, and other cardiovascular diseases.[qv: 1a,3] Most Mg^2+^ exists in bone, muscle, and soft tissues, and serum Mg^2+^ concentration (0.7–1.05 mmol L^−1^) cannot represent the total body Mg^2+^ content with taking only 1% of that.[qv: 1a,2c,g] Thus, the body can be in a state with severe Mg^2+^‐deficiency while the serum values could be within the normal range. According to this, medical influence of Mg^2+^‐deficiency could be largely underestimated. Moreover, Mg^2+^ levels in serum and other tissues are not routinely measured in clinical practices. Therefore, Mg^2+^ is often regarded as the “neglected” cation in human health.[qv: 1a]

Whether Mg^2+^ serves as a second messenger in intracellular signaling is controversial until recently.[qv: 2c,g] How extracellular Mg^2+^ promotes cellular activation signals is still not fully understood. Some reported Mg^2+^ transporters include transient receptor potential melastatin type 6/7 (TRPM6/7), Mg^2+^ transporter 1 (MagT1), solute carrier family 41 member 1/2 (SLC41A1/2), cyclin M2/3/4 (CNNM2/3/4), and mitochondrial RNA splicing 2 (MRS2).[qv: 1a] Some of these transporters are ubiquitously expressed whereas most are highly tissue, cell, and organelle specific. Mechanism of endothelial handling of Mg^2+^ is largely unknown although it plays a critical role in endothelial function and barrier integrity.[qv: 2d,3a]

The endothelial monolayer performs a pivotal role in regular tissue–fluid homeostasis, vascular tone, angiogenesis, and host defense.^4^ The endothelial barrier function and permeability are precisely regulated through cytoskeletal organization and junction proteins, which is also affected by many mediators including cytokines, growth factors, and ions. Inflammatory mediators including histamine, thrombin, bradykinin, vascular endothelial growth factor (VEGF), and angiopoietin 2 (Ang2) are shown to disrupt junctions and increase endothelial permeability.^4^ In contrast, factors like cyclic adenosine monophosphate (cAMP), Ras‐related protein 1 (Rap1), fibroblast growth factor (FGF), sphingosine‐1‐phosphate (S1P), Ras‐related C3 botulinum toxin substrate 1 (Rac1), and angiopoietin 1 (Ang1) are endothelial barrier‐stabilizing mediators.^4^ Maintaining the basal permeability and recovery after inflammatory events of endothelial barrier are largely dependent on its integrity and restoration, yet the molecular mechanisms are still not fully understood. In light of these findings, the goal in this study is to illustrate whether and how extracellular Mg^2+^ may regulate the endothelial barrier function.

Although the exact role of Mg^2+^ in different pathophysiological conditions remains elusive, Mg^2+^ has been used as a treatment for asthma, stroke, preeclampsia, and myocardial infarction in several large‐scale clinical trials over the last decade.[qv: 1a] Such therapeutic effects of Mg^2+^ are probably because Mg ion is a natural antioxidant, antiinflammation agent, and Ca^2+^/Na^+^ antagonist.^5^ It has also been well documented that Mg^2+^ can regulate vascular endothelial and smooth muscle cell functions related to hypertension and other cardiovascular diseases.^6^ Here, we showed some first evidence that Mg^2+^ may regulate endothelial barrier functions with the underlying mechanism by examining its effect on cell survival/growth, Ca^2+^ signaling, intracellular oxidative stress, endothelial permeability, cytoskeletal and junction protein organization, and endothelial barrier‐integrity mediators both in cellular models and Mg^2+^‐deficiency mouse model.

## 2 Results

### 2.1 Extracellular Mg^2+^ Concentration Affects its Transporters in a Dose‐Dependent Manner

**Figure 1:**
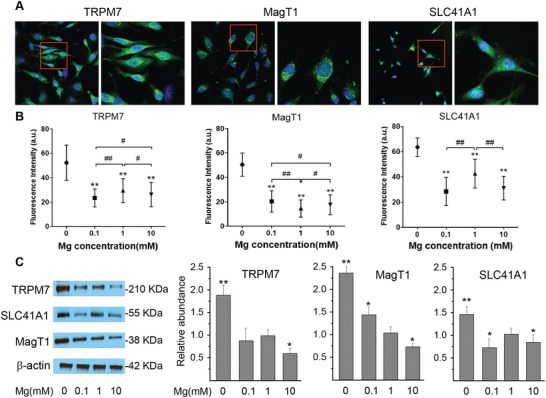
Extracellular Mg^2+^ concentration affects its transporters in a dose‐dependent manner. A) Representative confocal images showing cellular expressions of potential Mg transporters TRPM7, MagT1, and SLC41A1. Regions in red were enlarged in higher magnification. B) Effect of extracellular Mg concentrations on expression level of Mg transporters by quantitative fluorescent imaging analysis. (**P < 0.01, compare with 0 m; # *P* < 0.05, compare between groups; ## *P* < 0.01, compare between groups; *n* = 4). C) Western blot with quantitative analysis of TRPM7, MagT1, and SLC41A1 expression after exposure to a concentration gradient of Mg. (**P < 0.01, compare with 1 × 10^−3^ m; *P < 0.05, compare with 1 × 10^−3^ m; *n* = 3).

**Figure 2:**
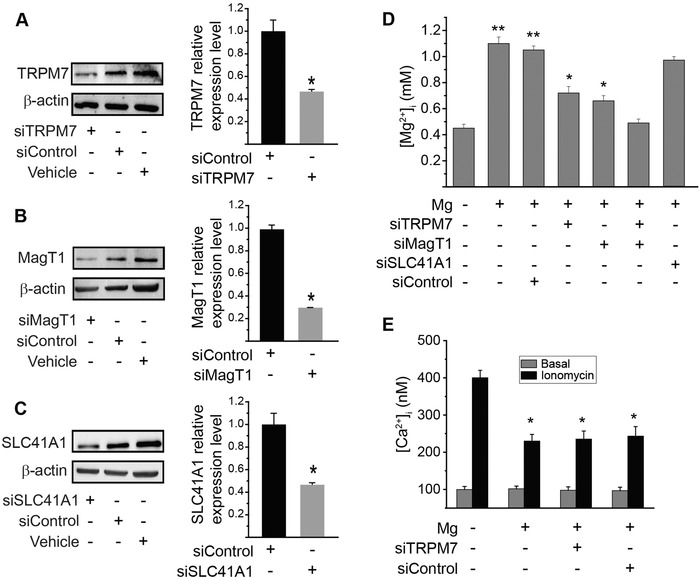
Intracellular Mg^2+^ concentration and Ca^2+^ influx in endothelium. A–C) siRNA knockdown of TRPM7, MagT1, and SLC41A1. Western blots showing expression of specific proteins in endothelial cells after treated with control siRNA or siRNA against TRPM7, MagT1, or SLC41A1; and quantitative measurements of relative protein levels from Western blots. (*P < 0.05, compared to control group; *n* = 4). D) Cells were transfected with siRNA against TRPM7, MagT1, SLC41A1, or scramble control siRNA, and were incubated with Mg^2+^‐free Ca^2+^‐containing medium for 15 min before being treated with high Mg^2+^ (2 mmol L^−1^ “+”) or Mg^2+^‐deficiency (0.1 mmol L^−1^ “–”). Mag fura‐2AM intensity was measured one minute after Mg^2+^ addition. (*P < 0.05, vs Mg^2+^‐deficiency or group treated with MagT1 siRNA and TRPM7 siRNA; **P < 0.01, vs Mg^2+^‐deficiency or group treated with MagT1 siRNA and TRPM7 siRNA; *n* = 3). E) Mg^2+^ inhibits Ca^2+^ influx in endothelium as an antagonist of Ca^2+^. Intracellular Ca^2+^ concentration measured by fura‐2AM intensity. Fura‐2AM intensity were recorded before ionomycin addition as basal level and one minute after ionomycin (10^−6^ mol L^−1^) addition. (* *P* < 0.05, vs Mg^2+^‐deficiency; *n* = 4).

Recent studies showed that Mg^2+^ influx can be controlled by various plasma membrane transporters including SLC41A1, MagT1, TRPM6, and TRPM7, and their expression might be tissue and cell specific.[qv: 1a,2e,g,3a,6b] We demonstrated previously that TRPM7 and MagT1 expressed in brain vasculatures.[qv: 3b] Here, we first showed the existence of SLC41A1, MagT1, and TRPM7 in vascular endothelial cells (**Figure** **1**A–C). Their expression level was also dependent on the extracellular concentrations of Mg^2+^.^7^ Generally, treatment of cell with decreased concentration of Mg led to increased expression of these transporters as demonstrated by both immunofluorescence imaging (Figure 1B) and Western blots (Figure 1C). Next, to determine which transporters are dominantly responsible for Mg^2+^ entry, we silenced the expression of these transporters using siRNA (**Figure** **2**A–C) and then measured intracellular Mg^2+^ level. Mg‐Fura2, an Mg^2+^ sensitive dye, was used to monitor intracellular Mg^2+^ concentration ([Mg^2+^]_i_) changes in cells. Data showed that the addition of Mg^2+^ into the Mg^2+^‐free bath induced a notable increase in [Mg^2+^]_i_, suggesting the entry of Mg^2+^ into cells. However, Mg^2+^‐entry was significantly inhibited after treatment with siRNA specifically against MagT1 and/or TRPM7, respectively, but not SLC41A1 (Figure 2C). Therefore, MagT1 and TRPM7 appear to be the plasma transporters for extracellular Mg^2+^ in vascular endothelium.

### 2.2 Mg^2+^ Inhibits Ca^2+^ Influx in Endothelium as an Antagonist of Ca^2+^

Mg^2+^ was reported to be an antagonist of Ca^2+^,[qv: 5b] so we evaluated if Mg^2+^ could attenuate the Ca^2+^ signaling in vascular endothelium by binding to this channel competitively. Ionomycin was used to induce a rapid intracellular Ca^2+^ concentration [Ca^2+^]_i_ rise (Ca^2+^ influx) as confirmed by increased fura‐2 fluorescence. Basal [Ca^2+^]_i_ was unchanged in all groups with different treatment. Mg^2+^ blocked the ionomycin‐mediated [Ca^2+^]_i_ responses significantly, indicating Mg^2+^ is an antagonist of Ca^2+^ signaling by binding to Ca^2+^ channels competitively (Figure 2E). TRPM7 is also a transporter for Ca^2+^, but knockdown of TRPM7 didn't block the [Ca^2+^]_i_ responses completely.

### 2.3 Mg^2+^ Suppresses Vascular Oxidative Stress

**Figure 3:**
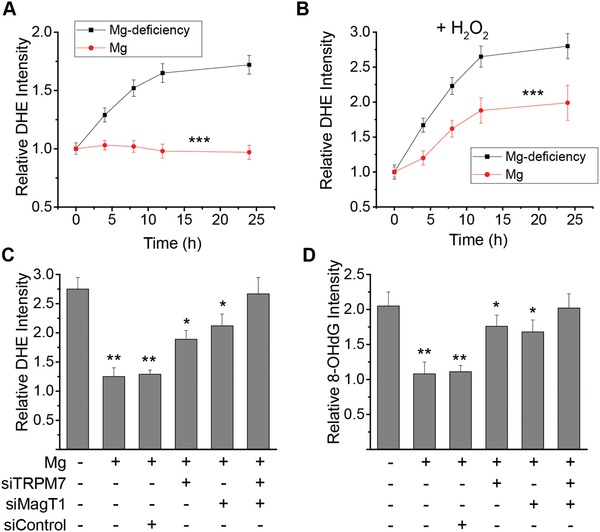
Mg^2+^ suppresses oxidative stress in endothelia. A) Time course of relative intracellular ROS level as compared to normal culture condition serving as control measured by DHE intensity with or without Mg^2+^ in endothelial cells. B) Time course of relative intracellular ROS level as compared to normal culture condition serving as control with the presence of extracellular H_2_O_2_ (50 µmol L^−1^) measured by DHE intensity with or without Mg^2+^ (10 × 10^−3^ m) in endothelial cells. C) Relative intracellular ROS level with the presence of extracellular H_2_O_2_ (50 × 10^−6^ m) measured by DHE intensity at 10 h post‐addition of Mg^2+^ (10 mmol L^−1^) or Mg^2+^‐deficiency (0.1 mmol L^−1^). D) Relative oxidative DNA damage as compared to normal culture condition serving as control with the presence of extracellular H_2_O_2_ (50 µmol L^−1^) measured by 8‐OHdG intensity at 10 h post‐addition of Mg^2+^ (10 mmol L^−1^) or Mg^2+^ deficiency (0.1 mmol L^−1^). (*P < 0.05, vs Mg^2+^‐deficiency or group treated with MagT1 siRNA and TRPM7 siRNA; **P < 0.01, vs Mg^2+^‐deficiency or group treated with MagT1 siRNA and TRPM7 siRNA; ***P < 0.001, vs Mg^2+^‐deficiency; *n* = 3).

Mg^2+^‐deficiency may be associated to oxidative stress.[qv: 5e,f] We, therefore, measured the intracellular reactive oxygen species (ROS) by DHE fluorescence after Mg^2+^ deprivation with or without H_2_O_2_ treatment. Mg^2+^‐deficiency induced significantly higher level of ROS over time regardless the presence of H_2_O_2_ (**Figure** **3**A,B). Mg^2+^‐treatment rescued the cells from oxidative stress, and a higher Mg^2+^ concentration (10 × 10^−3^ m) was required to significantly abolish the oxidative stress induced by addition of H_2_O_2_. MagT1 and/or TRPM7 knockdown by siRNA attenuated such rescue effect of Mg^2+^ on intracellular oxidative stress (Figure 3C). Similar results were observed on DNA oxidation as measured by 8‐OHdG intensity (Figure 3D).

### 2.4 Mg^2+^ Suppresses Vascular Inflammation

**Figure 4:**
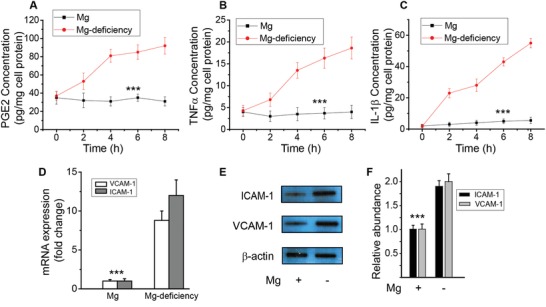
Mg^2+^ suppresses inflammation in endothelia. A–C) Effect of Mg^2+^‐deficiency on cytokines and pro‐inflammatory mediators release in endothelial cells. Time‐course release of PGE2, TNFα, and IL‐1β with Mg^2+^ (2 mmol L^−1^) or Mg^2+^‐deficiency (0.1 mmol L^−1^). D) Effect of Mg^2+^‐deficiency on endothelial cell adhesion molecules, the mRNA levels of ICAM‐1 and VCAM‐1, in animal models. E) Protein expression level of ICAM‐1 and VCAM‐1 by Western blot. F) Quantitative measurements of Western blot of ICAM‐1 and VCAM‐1. (***P < 0.001, vs Mg^2+^‐deficiency; *n* = 4).

Another hallmark of Mg‐deficiency in animal models is systemic inflammation,^8^ thus we examined the release profile of pro‐inflammatory factors and cytokines including PGE2, TNFα, and IL‐1β in endothelial cells using specific ELISA kits. Mg^2+^‐deficiency induced a significant time‐dependent release of these pro‐inflammatory factors (**Figure** **4**A–C). Next, we examined the effect of Mg^2+^‐deficiency on expression of cell adhesion molecules (i.e., ICAM‐1 and VCAM‐1), which are upregulated during vascular inflammation.[qv: 8a,c,9] In the Mg^2+^‐deficiency mouse model, the mRNA levels of ICAM‐1 and VCAM‐1 were significantly higher than that of normal controls in brain vasculature (Figure 4D). The protein expression of ICAM‐1 and VCAM‐1 in brain vascular tissues were also significantly upregulated in the Mg^2+^‐deficiency mouse model than in that of control (Figure 4E,F).

### 2.5 Mg^2+^ Regulates Endothelial Survival, Proliferation, and Motility through TRPM7 and MagT1

**Figure 5:**
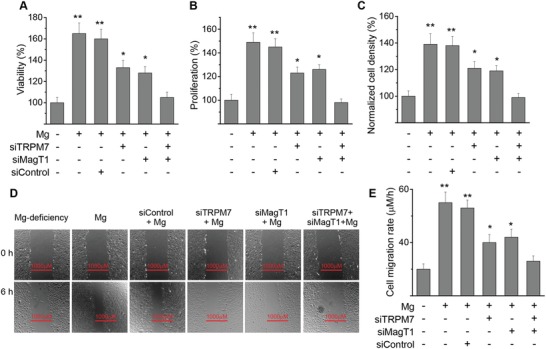
Mg^2+^ regulates endothelial survival, proliferation, motility and other cellular functions through TRPM7 and MagT1. A) Relative cell viability as compared to Mg^2+^‐deficiency (0.1 mmol L^−1^) measured by MTT assay. Cells were transfected with siRNA against TRPM7, MagT1, or scramble control siRNA. B) Relative cell viability as compared to Mg^2+^‐deficiency (0.1 mmol L^−1^) measured by BrdU assay. C) Relative cell adhesion density as compared to Mg^2+^‐deficiency (0.1 mmol L^−1^) measured 5 h after seeding. D) Representative images showing the cell migration treated with different siRNA with Mg or Mg^2+^‐deficiency conditions using scratch assay. E) Cell migration rate measured 24 h after seeding with or without Mg^2+^. (*P < 0.05, vs Mg^2+^‐deficiency or group treated with MagT1 siRNA and TRPM7 siRNA; **P < 0.01, vs Mg^2+^‐deficiency or group treated with MagT1 siRNA and TRPM7 siRNA; *n* = 4).

To verify if Mg^2+^ is an essential element for endothelial survival and growth, we measured cell viability and proliferation. Cell viability and proliferation with Mg^2+^‐deficiency was significantly lower than that with Mg^2+^‐treatment (**Figure** **5**A,B). Similar results were observed on cell adhesion and migration (Figure 5C–E). MagT1 and/or TRPM7 suppression by siRNA reduced such rescue effect of Mg^2+^‐treatment on endothelial cells.

### 2.6 Mg^2+^ Regulates Endothelial Barrier Integrity through S1P1‐Rac1 Pathways

**Figure 6:**
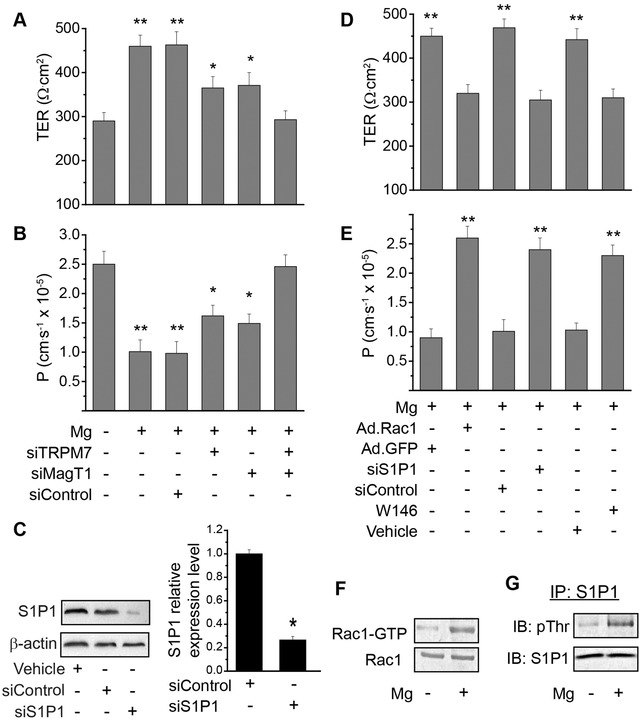
Mg^2+^ regulates endothelial barrier integrity through S1P1‐Rac1. A) TER measurements with the treatment of Mg^2+^ (2 mmol L^−1^) or Mg^2+^‐deficiency (0.1 mmol L^−1^). Cells were transfected with siRNA against TRPM7, MagT1 or scramble control siRNA, and cultured to confluency in transwell. B) Trans‐endothelial permeability to dextran (40 kD) measurements with the treatment of Mg^2+^ (2 mmol L^−1^) or Mg^2+^‐deficiency (0.1 mmol L^−1^). C) siRNA knockdown of S1P1. Western blots showing protein expression of S1P1 in endothelial cells after treated with control siRNA or siRNA against S1P1; and quantitative measurements of relative S1P1 protein levels from Western blots (right). D) TER measurements with the treatment of Mg^2+^ (2 mmol L^−1^) in cells with downregulation of Rac1 or S1P1 using siRNA or S1P1 specific inhibitor W146. E) Trans‐endothelial permeability to dextran (40 kD) with the treatment of Mg^2+^ (2 mmol L^−1^) in cells with downregulation of Rac1 or S1P1 using siRNA or S1P1 specific inhibitor W146. F) Rac1‐GRP activity with or without Mg^2+^ treatment. G) Phosphorylation of S1P1 with or without Mg^2+^ treatment. *P < 0.05, vs Mg^2+^‐deficiency or group treated with MagT1 siRNA and TRPM7 siRNA. (*P < 0.05, vs Mg^2+^‐deficiency or group treated with MagT1 siRNA and TRPM7 siRNA; **P < 0.01, vs Mg^2+^‐deficiency or group treated with MagT1 siRNA and TRPM7 siRNA; *n* = 4).

Endothelial barrier integrity was measured by TER and permeability to Dextran (40 kDa). Significantly lower TER and higher permeability were observed after Mg^2+^‐deficiency compared to that with Mg^2+^ (**Figure** **6**A,B). To understand the molecular mechanisms that may mediate the effect of Mg^2+^ on endothelial barrier integrity, we explored the involvement of S1P1‐Rac1 pathways as suggested by our previous studies.^10^ Results showed that Mg^2+^‐regulated endothelial integrity could be significantly blocked by suppression of S1P1 via siRNA (Figure 6C) or specific inhibitor against S1P1, or Rac1 using dominant negative adenovirus against Rac1 (Figure 6D,E). It was also verified that Mg^2+^ included significant activation of S1P1 and Rac1 via phosphorylation (Figure 6F,G).

### 2.7 Mg^2+^ Regulates Endothelial Barrier‐Stabilizing Mediators—cAMP, FGF1, FGF2, VEGFA, and eNOS

**Figure 7:**
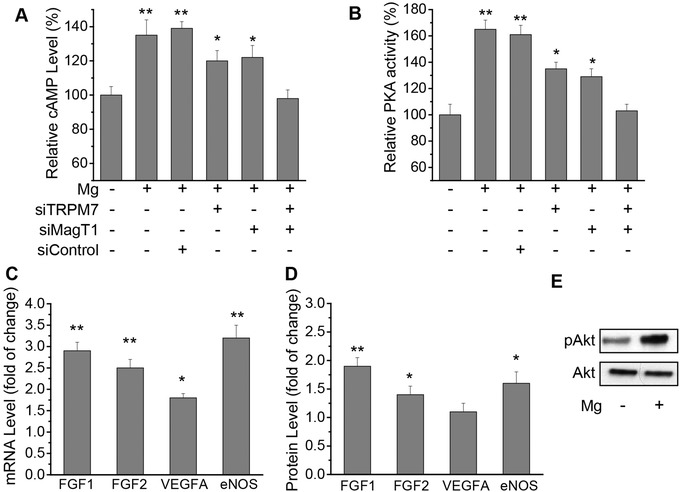
Mg^2+^ regulates endothelial barrier‐stabilizing mediators. A) Relative intracellular cAMP level as compared to Mg^2+^‐deficiency (0.1 mmol L^−1^) measured 1 h after Mg^2+^ (2 mmol L^−1^) addition. B) Relative intracellular PKA activity as compared to Mg^2+^‐deficiency (0.1 mmol L^−1^) measured 1 h after Mg^2+^ (2 mmol L^−1^) addition. C) Relative mRNA level of key endothelial barrier permeability mediator FGF1, FGF2, VEGFA, and eNOS as compared to Mg^2+^‐deficiency (0.1 mmol L^−1^) measured 24 h after Mg^2+^ (2 mmol L^−1^) addition. D) Relative protein expression level of key endothelial barrier permeability mediator FGF1, FGF2, VEGFA, and eNOS as compared to Mg^2+^‐deficiency (0.1 mmol L^−1^) measured 24 h after Mg^2+^ (2 mmol L^−1^) addition. E) Phosphorylation of Akt with or without Mg^2+^ treatment. (* *P* < 0.05, vs Mg^2+^‐deficiency or group treated with MagT1 siRNA and TRPM7 siRNA; ** *P* < 0.01, vs Mg^2+^‐deficiency or group treated with MagT1 siRNA and TRPM7 siRNA; *n* = 3).

Barrier integrity and stability is critical for maintenance of endothelial functions, yet the molecular mechanisms are largely unknown. Some known mediators of barrier stability include S1P and Rac1, as already demonstrated by our data shown above (Figure 6). We next examined the intracellular cAMP level and the activation of PKA pathway because they are also the important endothelial barrier‐stabilizing mediators and modulators for cell viability.^11^ Cellular cAMP level and PKA activity with Mg^2+^‐deficiency were significantly lower than that of Mg^2+^‐treatment (**Figure** **7**A,B). MagT1 and/or TRPM7 suppression by siRNA attenuated the rescue effect of Mg^2+^‐treatment. Our results also showed that expression of key transendothelial permeability regulators including FGF1, FGF2, VEGFA, and eNOS was significantly enhanced at mRNA level revealed by RT‐PCR after Mg^2+^ treatment, as compared to Mg^2+^‐deficiency (Figure 7C). Similar results were observed at protein level revealed by ELISA except for VEGFA (Figure 7D). We also showed significantly enhanced activation of Akt by phosphorylation with Mg^2+^‐treatment (Figure 7E), consistent with the notion that Akt‐mediated eNOS activation leads to enhanced vascular stability.^12^

### 2.8 Mg^2+^ Regulates Endothelial Cytoskeletal Reorganization and Junction Proteins

**Figure 8:**
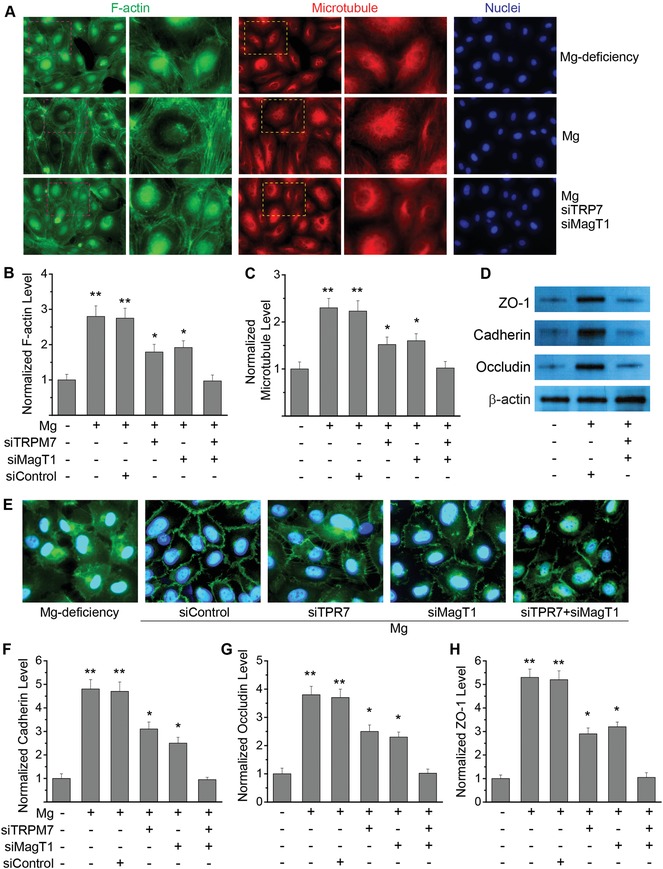
Mg^2+^ regulates endothelial cytoskeletal reorganization and junction proteins. A) Representative fluorescent images of endothelial cells stained with F‐actin (green), microtubule (red), and nuclei (blue) with Mg^2+^‐deficiency (0.1 mmol L^−1^) and Mg^2+^‐treatment (2 mmol L^−1^) with or without siRNA against both MagT1 and TRPM7. Regions in red or yellow were enlarged in higher magnification. B) Quantitative measurements of normalized F‐actin level in each cell as compared to Mg^2+^‐deficiency with or without siRNA against MagT1 and/or TRPM7. C) Quantitative measurements of normalized microtubule level in each cell as compared to Mg^2+^‐deficiency with or without siRNA against MagT1 and/or TRPM7. D) Western blot analysis showing the expression of cadherin, occluding and ZO‐1 with Mg^2+^‐deficiency (0.1 mmol L^−1^) and Mg^2+^‐treatment (2 mmol L^−1^) with or without siRNA against both MagT1 and TRPM7. E) Representative fluorescent images of endothelial cells stained with cadherin (green) and nuclei (blue) with Mg^2+^‐deficiency and Mg^2+^‐treatment with siRNA against MagT1 and/or TRPM7. F) Quantitative measurements of normalized cadherin level in each cell as compared to Mg^2+^‐deficiency with or without siRNA against MagT1 and/or TRPM7. G) Quantitative measurements of normalized occludin level in each cell as compared to Mg^2+^‐deficiency with or without siRNA against MagT1 and/or TRPM7. H) Quantitative measurements of normalized ZO‐1 level in each cell as compared to Mg^2+^‐deficiency with or without siRNA against MagT1 and/or TRPM7. (*P < 0.05, vs Mg^2+^‐deficiency or group treated with MagT1 siRNA and TRPM7 siRNA; **P < 0.01, vs Mg^2+^‐deficiency or group treated with MagT1 siRNA and TRPM7 siRNA; n = 4).

Cytoskeletal organization and junction proteins are the ultimate structures controlling the endothelial barrier functions. Thus, we examined how Mg^2+^ would affect their expression and organization. Compared to Mg^2+^‐deficiency, Mg^2+^‐treatment induced significant cytoskeletal reorganization as more F‐actin and microtubule presented inside cells, and were distributed more toward the cell edge forming a cobble‐stone shape (**Figure** **8**A–C), indicating a more tightened endothelial barrier. Moreover, Mg^2+^‐treatment stimulated significantly higher expression of junction proteins including VE‐cadherin, occluding, and zonula occludens‐1 (ZO‐1) compared to Mg^2+^‐deficiency (Figure 8D–H). MagT1 and/or TRPM7 suppression by siRNA reduced such effect of Mg^2+^ on cytoskeleton and junction proteins.

### 2.9 Mg^2+^‐Deficiency Enhances Endothelial Barrier Permeability In Vivo

**Figure 9:**
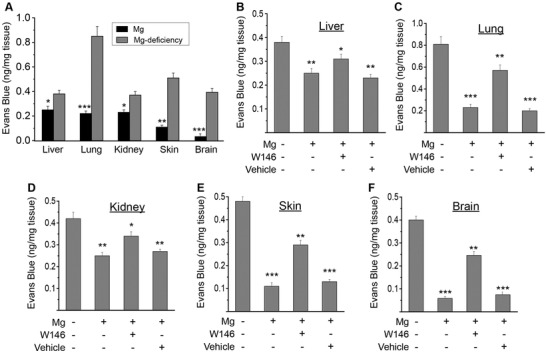
Mg^2+^‐deficiency enhances endothelial barrier permeability in vivo. A) Quantitation of Evans Blue extravasation in different tissues of Mg^2+^‐deficiency mouse and control mouse (Mg^2+^‐treatment). B) Quantitation of Evans Blue extravasation in liver. C) Quantitation of Evans Blue extravasation in lung. D) Quantitation of Evans Blue extravasation in kidney. E) Quantitation of Evans Blue extravasation in skin. F) Quantitation of Evans Blue extravasation in brain. Normal mouse was treated with histamine to induce transient vessel hyper‐permeability, and may also be subjected to Mg^2+^‐treatment with or without S1P1 antagonist W146. (* *P* < 0.05, vs Mg^2+^‐deficiency; ** *P* < 0.01, vs Mg^2+^‐deficiency; *** *P* < 0.001, vs Mg^2+^‐deficiency; *n* = 6).

Mg^2+^‐deficiency mouse model was created by feeding with Mg^2+^‐deficiency diet as demonstrated by previous studies.^13^ Vessel permeability in different organs was examined using Miles assay.^14^ Data showed that Mg^2+^‐deficiency caused significant leakage in blood vessels in different organs including liver, lung, kidney, skin, and brain, with lung, skin, and brain as the most severe ones (**Figure** **9**A).

### 2.10 Mg^2+^‐Treatment Rescues Mouse from Histamine‐Induced Transient Vessel Hyper‐Permeability

Transient vessel hyper‐permeability in mouse was created with histamine treatment.^15^ Compared to Mg^2+^‐deficiency, Mg^2+^‐treatment significantly reduced the hyper‐permeability induced by histamine in vessels of liver, lung, kidney, and skin and brain tissues (Figure 9B–F). Block of S1P1 with its specific antagonist W146 attenuated this rescue effect of Mg^2+^ on vessel permeability in mouse.

## 3 Discussion

Mg^2+^ is essential for many of the cells and organs to function because it plays a crucial role in human physiology. It is required for the structure of bones, acts as a cofactor for >300 enzymes, DNA and RNA, as well as ATP, and affects permeability of membranes and neuromuscular transmission. Despite its irreplaceable roles, little is known about Mg^2+^ physiology and homeostasis. Nutritionists believe that the daily intake of Mg through the diet perhaps is not sufficient for the general population.[qv: 3a] The effects of hypomagnesemia are for the most part undetected. Simple, widespread assessment of Mg^2+^ intake remains unavailable for humans. Most of the patients admitted to clinics are unaware of their low Mg^2+^ levels. In addition, serum Mg^2+^ levels could be a poor predictor of tissue Mg^2+^ levels because of its predominant presence as an intracellular cation (>99%).[qv: 3a] Due to its importance to human body, Mg^2+^ plays an essential role in disease precaution, treatment, and entire health. Hypomagnesemia has been linked to many chronic disorders such as Alzheimer's disease, Parkinson's disease, stroke, hypertension, migraine headaches, cardiovascular abnormalities, and diabetes.[qv: 1b]

Many cellular processes like the synthesis of DNA and protein are highly dependent on the availability of intracellular Mg^2+^, thus intracellular concentrations of Mg^2+^ are strictly adjusted.[qv: 1a] Demonstrating the molecular properties of transporters involved in Mg^2+^ homeostasis has been the main target of research over the last two decades. A few Mg^2+^ transporters have been identified through genetic screenings on human diseases. The exact role of these transporters need further investigations and several of them have been characterized functionally.[qv: 1a,2d] TRPM7, MagT1, SLC41A1, and CNNM3 are ubiquitous and expressed on plasma membrane. SLC41A2 is mainly on Golgi membrane while MRS2 is on mitochondrial membrane though they are also ubiquitously expressed. TRPM6 is primarily located in epithelial cells in kidney and intestine. CNNM1 is found to be mainly in brain tissue. CNNM2 and CNNM4 are primarily located on the basolateral plasma membrane of kidney and intestine, respectively.[qv: 1a,2d] The specific Mg^2+^ transporters responsible for Mg^2+^ homeostasis in endothelium is still unknown. In this study, we found that TRPM7 and MagT1 are the major plasma membrane transporters facilitating extracellular Mg^2+^‐entry into cells and regulating subsequent Mg^2+^‐signaling.

Mg^2+^ is a known natural antioxidant, and reduces inflammation in various pathological conditions.[qv: 5a,c,d,16] Therefore, it has protective effect on different cells including neurons and vascular cells due to inflammation and oxidation. For example, MgSO_4_ treatment reversed lipopolysaccharide‐mediated inhibition of apolipoprotein E mRNA expression in both rat and human placentas.[qv: 5a] Oral Mg^2+^ supplementation induces favorable antiatherogenic changes in apolipoprotein E‐deficient mice.^17^ More interestingly, Mg^2+^ could overcome the deleterious metabolic consequences related to apolipoprotein E4.^18^ In addition, Mg^2+^‐deficiency induced a decrease in the percent composition of apolipoprotein E in rats.[qv: 13a] Consistent with these findings, our data also demonstrated that Mg^2+^ can suppress ROS production in endothelium with or without pro‐oxidant H_2_O_2_.

Acting like a natural Ca^2+^ channel blocker and competing with Na^+^ for binding sites on cells are one of the mechanisms that Mg^2+^ protects cells.[qv: 5b] Thus, it decreases intracellular Ca^2+^ and Na^+^, binds to K^+^ in a cooperative manner, increases prostaglandin E level, improves endothelial dysfunction, induces endothelial‐dependent vasodilation, and reduce blood pressure.[qv: 6c] K^+^ is vasoactive and increases blood flow when infused into the arterial supply of a vascular bed. The vasodilation results from hyperpolarization of the vascular smooth muscle cell subsequent to K^+^ stimulation by the ion of the electrogenic Na^+^–K^+^ pump and/or activating the inwardly rectifying K^+^ channels.^19^ Intracellular Mg^2+^ can regulate this rectification through blocking the K^+^ channel, functioning in at least four potassium channels.^20^ It can also regulate the current through K^+^ channels in an outward direction.^21^ Thus, Mg may potentially regulate blood flow through K^2+^ channels which become more prominent when going down from conduit arteries to resistance vessels embedded in parenchyma.^22^ Mg was shown to be more effective than nifedipine in improving blood flow in the ischemic area of the brain.^23^ Besides regulating intracellular Ca^2+^, Na^+^, and K^+^, Mg^2+^ can control cellular pH, insulin sensitivity, left ventricular mass, as well as arterial compliance. It also holds back circulating Na^+^/K^+^‐ATPase suppressive activity that decreases vascular tone.[qv: 6c] Mg^2+^, as an analogous Ca^2+^ channel blocker, results in formation of vasodilator prostacyclin and NO, and alters the vascular reaction to vasoactive agonists.[qv: 1b,3a,5b,6c] These biochemical processes administer vascular contraction and relaxation, survival and growth, differentiation, and inflammation. Variation in Mg^2+^ hemostasis and transport system may predispose patients to hypertension and other cardiovascular dysfunctions.

Another mechanism by which Mg^2+^ controls the endothelial barrier function is by regulating barrier‐stabilizing mediators. Results showed Mg^2+^ enhanced the protein expression of cAMP, FGF1/2, Akt, Ang, and eNOS. Upregulation of cAMP levels by Mg^2+^ diminishes vascular leakage via activating PKA, in line with a previous study.^24^ It is shown that cAMP activates Rap1, leading to F‐actin reorganization and enhanced junctional adhesions. Latest research on Rap1 claims that it forms a cooperative association with VE‐cadherin, due to the ability of modulating each other's responses.^25^ More recently, FGF has been found to play an important role in junction integrity, consistent with our observations. Lack of FGF signaling may reduce the level of the protein‐tyrosine phosphatase 1D, leading to enhanced VE‐cadherin phosphorylation and damaging its binding to p120 catenin. It is well documented that VE‐cadherin improves barrier stability by limiting growth factor signaling, such as VEGF, transforming growth factor β, and platelet‐derived growth factor that increase the permeability of endothelial barrier following angiogenic responses.^25^, ^26^ Sphingosine‐1‐phosphate (S1P), another powerful barrier‐stabilizing factor, binds to S1P1 to cause reorganization of cortical actin through a serial of downstream targets, including Rac1, cortactin, focal adhesion kinase, actinin, as well as paxillin.^27^ Blockade of the S1P1 signaling can lead to junction destabilization, permeability, and aberrant angiogenesis in vivo. Moreover, Ang as well as their Tie receptors are important modulators of vascular maturation and quiescence. One interesting feature of Ang1 signaling is that it functions through the same receptor in both vascular remodeling and quiescence.^12^ Emerging evidence claims that cellular contacts between endothelial cells plays a decisive role in Ang1‐mediated differential Tie‐2 localization and signaling. Upon Ang1 exposure, homotypic cell‐cell contacts between endothelial cells will induce recruitment of Tie2 to cell interactions, resulting in enhanced vascular stability following Akt‐mediated eNOS phosphorylation.^12^ This is also in line with our findings on the regulation of Ang1, Akt, and eNOS by Mg^2+^ to enhance endothelial barrier functions.

## 4 Conclusion

**Figure 10:**
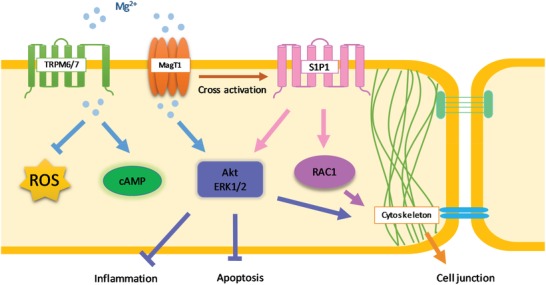
Schematic chart showing the molecular pathway for Mg^2+^‐regulated endothelial barrier function. Extracellular Mg ion enters cells through TRPM7 and MagT1 which may cross activate S1P1 which, in turn, activates Akt, ERK1/2, and Rac1. Intracellular Mg^2+^ signaling may lead to the increased cAMP and PKA activity which is important for cell survival and growth. Activated Akt and ERK1/2 will also suppress inflammation and apoptosis. In addition, Mg^2+^ suppresses the production of ROS as a nature antioxidant. Akt, ERK1/2 and Rac1 signaling will eventually induce cytoskeletal reorganization, enhanced junction protein expression and polarized distribution, leading to tightened endothelial barrier.

In summary, this study provides novel insight showing that Mg^2+^ regulates the endothelial barrier function through TRPM7, MagT1, and S1P1‐Rac1 pathways both in vitro and in vivo (**Figure** **10**). Barrier stabilizing‐mediators and junction proteins are also targets of regulation for Mg^2+^ to enhance endothelial integrity. Mg^2+^‐deficiency could be the causing factor for many pathological conditions. This work could open up a new avenue to use Mg as therapeutic agent to treat some vascular diseases related to endothelial barrier functions.

## 5 Experimental Section

*Cell Viability and Proliferation*: Endothelial cell viability was measured by 3‐(4,5‐dimethylthiazol‐2‐yl)‐2,5‐diphenyltetrazolium bromide (MTT) assay as described before.[qv: 6k,28] Cell proliferation was measured using a BrdU cell proliferation kit as described before.[qv: 6k,28]

*Intracellular ROS Measurement*: Dihydroethidium (DHE) was used to determine reactive oxygen species production following a modified protocol as described before.^29^ Oxidative DNA damage was measured by detection of 8‐hydroxy‐deoxyguanine (8‐OHdG) coupled with diaminobenzidine (DAB) as described before.[qv: 5f]

*Cell Adhesion and Migration*: Cell adhesion and migration assays were carried out as described before.[qv: 6k] The average cell migration rate was calculated.

*cAMP and PKA Activity*: Cellular cAMP activity was measured using a direct immunoassay kit following manufacture's instruction.^11^ PKA activation was determined using PKA kinase activity assay kit (ab139435, Abcam, USA) by following manufacture's protocol.^11^

*Real Time PCR*: After different treatments, cells were harvested and total RNA was extracted by using an RNeasy Mini kit (Qiagen, US) and subsequently quantified using a spectrophotometer (Nanodrop 2000, US) with OD260/OD280 ratios between 1.9 and 2.1. Real time PCR was used to estimate the mRNA levels of different gene expression in a CFX96 Touch RT‐PCR Detection System (Bio‐Rad, US). Data was analyzed by Bio‐Rad CFX Manager 3.1 (Biorad, US). The 2−ΔΔCt method was used to calculate gene fold changes. The level of specific mRNA was normalized to the endogenous control, β‐actin.[qv: 6e,30]

*Gene Silencing*: Small interference RNA was used to knockdown specific gene expression. Briefly, cells were transfected with siRNA using Lipofectamine 2000 (Invitrogen, CA, US) according to the manufacturer's protocol, and used 48 h post transfection. Small interfering RNA (siRNA) targeting human TRPM7, MagT1, and S1P1 were purchased from Santa Cruz Biotechnology. The corresponding scrambled siRNAs were used as controls.^10^

*Transmonolayer Electrical Resistance (TER)*: The cells were cultured on inserts with collagen‐coated polycarbonate in transwell, and TER was measured using an Endohmeter (World Precision Instruments, Sarasota, FL) as described before.^10^

*Measurement of [Mg^2+^]_I_ and [Ca^2+^]_i_*: Mag fura‐2AM and fura‐2AM, working as selective fluorescent probes, were used to measure [Mg^2+^]_I_ and [Ca^2+^]_I_, respectively, as previously described in the article.[qv: 6b] [Mg^2+^]_I_ responses to increasing concentrations of extracellular Mg^2+^ (2 mmol L^−1^) were measured in cells incubated in Mg^2+^‐free, Ca^2+^‐containing modified Hanks' buffer. Cells were exposed to Mg^2+^‐free buffer for 15 to 20 min before addition of extracellular Mg^2+^. [Ca^2+^]_I_ reaction to ionomycin (10^−6^ mol L^−1^) were performed in cells incubated for 15 to 20 min in Ca^2+^‐containing modified Hank's buffer with Mg^2+^ (1.3 mmol L^−1^).

*Permeability of the Endothelial Barrier*: Fluorescein‐isothiocyanate (FITC)‐labeled dextran (40 kDa, Invitrogen) was used to measure endothelial permeability in transwells as described before.^10^

*Protein Expression and Phosphorylation*: Protein expression and phosphorylation were determined by Western blot as described before.^10^, ^29^ Briefly, extracted protein of interest was separated by gel electrophoresis and transferred to PVDF membranes. Membranes were blocked and incubated overnight with specific antibodies against proteins of interest. ELISA kits (Thermo Fisher) were also used to determine some secreted proteins of interest in endothelial cells, including FGF1, FGF2, VEGFA, and eNOS as well as PGE2, TNFα, and IL‐1β by following manufacture's protocols.

*Animal Model and In Vivo Blood Vessel Permeability Measurement Using Miles Assay*: All procedures were approved by institutional IACUC following NIH guidelines for the care and use of laboratory animals. C57 mice of 8–10 weeks old (Jackson Labs) were selected for equal age and weight (*n* = 6). Additionally, equal numbers of male and female mice were used, thus sex was not a biological variable in this specific study. Mg‐deficient mouse model was created by an Mg‐deficient diet for 15 days.^13^ The Mg concentrations of diets were 60 mg kg^−1^ and 1,000 mg kg^−1^ for Mg‐deficient and control diets, respectively. The intravenous injection of Evans Blue in mouse animal model is a fundamental step of Miles assay.^14^ Evans Blue is a dye that binds albumin. The endothelium is impermeable to albumin under normal conditions, therefore Evans Blue bound albumin remains restricted within blood vessels. Under pathologic conditions, small proteins such as albumin can permeate the endothelium, which permits the extravasation of Evans Blue in tissues. Thus, a normal endothelium keeps away the extravasation of dye in the nearby vascularized tissues while organs with impaired permeability will show significantly increased blue coloration. The level of vascular permeability can be assessed by quantitative measurement of the dye. When ready for in vivo experiments, mice were put inside a cramped device so that the animal cannot freely move but its tail can be handled. Briefly, 200 µL 0.5% sterile solution of Evans Blue in PBS was intravenously injected into the mouse lateral tail vein. After 30 min, mice were sacrificed by cervical dislocation. To meet the demand of Miles assay, cervical dislocation is suggested as it limits significant interference with vascular permeability. Organs of interest were then collected, air dried, and incubated in formamide at 55 °C for 48 h to extract Evans Blue from tissues. After centrifugation, solutions were subjected to absorbance measurement at 610 nm. The amount of Evans Blue (ng) extravasated per mg tissue was then calculated.

*Mouse Brain Vessels Isolation and Expression of Cell Adhesion Molecules*: Isolation of whole mouse cerebrovascular fragments was performed as described.^31^ Vascular tissues were then homogenized and cell lysates were collected for cell adhesion protein ICAM‐1 and VCAM‐1 expression using Western blot.

*Transient Vessel Permeability Increase and Mg Treatment In Vivo*: To evaluate the rescue effect of Mg^2+^ on endothelial permeability in vivo, a transient leaky vessel model in mice was created by histamine treatment with or without Mg^2+^.^15^ Similar to the Miles assay, C57 mice of 8–10 weeks old (Jackson Labs) were selected for equal age and weight, and same numbers of male and female mice were used, thus sex was not a biological variable in this specific study as well (*n* = 6). Mice were first pretreated with Mg^2+^ or saline control. MgSO_4_ injection was administrated by an IP dose of 350 mg kg^−1^ followed by 50 mg kg^−1^ every 20 min for 4 h; injections were given in a volume of 0.1 mL. A second dose of 350 mg kg^−1^ was given at the end of the 4 h period. Mice were then injected with Evans Blue solution. To induce vascular hyper‐permeability, 5 mg histamine (Sigma‐Aldrich) dissolved in 500 µL PBS was injected via tail vein. After 30 min, mice were sacrificed and subjected to Miles assay. The S1P1 specific inhibitor W146 on Mg‐mediated endothelial integrity was also assessed by pretreating mouse with W146 (20 mg kg^−1^) together with MgSO_4_.

*Statistical Analysis*: All data were presented as mean ± standard deviation. Each cellular experiment was repeated independently minimal of three time (*n* = 3), and each in vivo study was performed using six animals (*n* = 6). One‐way or two‐way analysis of variance (ANOVA) followed by Tukey post hoc test was used to determine statistically significant differences. Unpaired Student *t*‐test was also used to compare difference between two groups as appropriate. *P* < 0.05 was considered statistically significant.

## Conflict of Interest

The authors declare no conflict of interest.

## Supporting information

Supplementary.
