# Supplementary material for: Magnesium Regulates Endothelial Barrier Functions through TRPM7, MagT1, and S1P1
Source: Adv Sci (Weinh). 2019 Jul 30;6(18):1901166. doi: 10.1002/advs.201901166 (PMC6755513; doi:10.1002/advs.201901166)
Supplement: Supplementary file 1 — Supplementary. [file ADVS-6-1901166-s001.pdf]

Copyright WILEY-VCH Verlag GmbH & Co. KGaA, 69469 Weinheim, Germany, 2019.

ADVANCED SCIENCE
Open Access

Supporting Information

for *Adv. Sci.,* DOI: 10.1002/advs.201901166

Magnesium Regulates Endothelial Barrier Functions through TRPM7, MagT1, and S1P1

Donghui Zhu, Jing You, Nan Zhao, and Huaxi Xu*

Supporting Information

Magnesium Regulates Endothelial Barrier Functions through TRPM7, MagT1 and S1P1

Donghui Zhu, Jing You, Nan Zhao, Huaxi Xu*

Prof. D. Zhu
Department of Biomedical Engineering, Institute for Engineering-Driven Medicine, College of Engineering and Applied Sciences, Renaissance School of Medicine, Stony Brook University, Stony Brook, NY 11794, USA
E-mail: donghui.zhu@unt.edu

J. You
Department of Biomedical Engineering, University of North Texas, Denton, TX 76207, USA

N. Zhao
Department of Biomedical Engineering, Pennsylvania State University, State College, PA 16802, USA

Prof. H. Xu
Sanford Burnham Prebys Medical Discovery Institute, La Jolla, CA 92037, USA

Detailed Methods

Chemicals and Reagents

MTT (V13154, Invitrogen, Carlsbad, CA, US), BrdU cell proliferation kit (6813, cell signaling biotechnology, Danvers, MA, US), DHE (D1168, Invitrogen, Carlsbad, CA, US), RNeasy Mini Kit (74104, Qiagen, Germantown, MD, US), Lipofectamine 2000 (11668027, Invitrogen, Carlsbad, CA, US), TRPM7 polyclonal antibody (PIPA515302, Invitrogen, Carlsbad, CA, US), MagT1 polyclonal antibody (PIPA562063, Invitrogen, Carlsbad, CA, US), SLC41A1 polyclonal antibody (PIPA553286, Invitrogen, Carlsbad, CA, US), Goat anti rabbit Alexa Fluor 488 polyclonal secondary antibody (A11008, Invitrogen, Carlsbad, CA, US), siRNA targeting TRPM7 (Santa cruz biotechnology, Dallas, TX, US), siRNA targeting MagT1(sc-91352, Santa cruz biotechnology, Dallas, TX, US), siRNA targeting SLC41A1 (sc-88707, Santa cruz biotechnology, Dallas, TX, US), control siRNA (sc-37007, Santa cruz biotechnology, Dallas, TX, US), Mag fura-2AM (M1291, Invitrogen, Carlsbad, CA, US), fura-2AM (F1201, Invitrogen, Carlsbad, CA, US), Mg free, Ca containing Hanks' Buffer (AAJ67681K2, Alfa Aesar, Haverhill, MA, US), Mg-free medium (D9785, Sigma-Aldrich Corp, St. Louis, MO, US), Ionomycin (ICN1550711, MP Biomedicals, Santa Ana, CA, US), PKA kinase activity assay kit (ab139435, Abcam, US), fluorescein-isothiocyanate (FITC)-labeled dextran (40 kDa, Invitrogen), histamine (Sigma-Aldrich Corp, St. Louis, MO, US).

Solutions preparation

$MgSO_4$ solutions were prepared by dissolving $MgSO_4$ (Sigma Aldrich, US) into deionized water. Solutions were filtered by 0.22 μm filter (Thermo Fisher Scientific, US) and autoclaved (Harvey

Sterile Max, Thermo Scientific, US). Different concentrations of $MgSO_4$ solutions were prepared by diluting with Mg^{2+} free cell culture medium (Sigma Aldrich, US). [1]

Cell culture

Primary human coronary artery endothelial cell (HCAEC) was ordered from ATCC. The culture medium for HCAECs (ECM, ScienCell, US) was basal medium, 5% fetal bovine serum, 1% endothelial cell growth supplement, and 1% penicillin/streptomycin. HCAECs were maintained in flask (Falcon, BD Bioscience, US) until ~ 90% confluence was reached. Then cells were detached by trypsin/EDTA (Life technologies, US), centrifuged (Sorvall Biofuge Stratos, Thermo Electron Corporation, US), and resuspended. The cell number was counted by cell counter (Bio-Rad, US) before subculture. Low passage cells (passages 2 to 5) were studied.[1-2]

Cell viability and Proliferation

Cells were seeded in the 96-well cell culture plate (BD Biosciences, US) with 5000 cells per well for 24 hours to allow cell attachment. Medium was replaced by medium supplemented with different ion solutions and incubated for 24 hours. Medium with 10% DMSO (Life Technologies, US) and medium alone were positive and negative controls. Another blank reference containing the same concentration of ion solution without cells was used to exclude the interference of the ions. The 3-[4,5-dimethylthiazol-2-yl]-2,5-diphenyltetrezolium bromide (MTT, Invitrogen, US) test was performed according to the manufacturer's protocol. Absorbance (A) was measured at 570 nm using a microplate reader (SpectraMax, Molecular Devices, US). A BrdU cell proliferation kit (Cell Signaling, US) was used for the cell proliferation test. Cells were seeded in a 96-well cell culture plate at 5,000 cells per well. After 24 hours, medium was replaced by

different ion solutions and incubated for 24 hours. The ion concentration was set up to the concentration at which cell viability was not significantly affected. The proliferation test was performed according to manufacturer's protocol. Absorbance was measured at 450 nm. Positive control and negative control were medium without ion supplement and medium without cells.[1-2]

Measurement of superoxide anion production

We used DHE (dihydroethidium) to determine superoxide anion production following a modified protocol as described before.[3] Upon contact with superoxide anions, oxoethidium, a highly fluorescent product from the oxidative reaction of DHE, binds to DNA, causing an increase in the fluorescence intensity of the cell nuclei. In the present study, cells were grown in eight-well chambers and serum-starved overnight. Cells were rinsed twice with warm serum-free and Phenol Red-free DMEM and incubated with DHE (5 μM) in Phenol Redfree DMEM at 37°C for 30 min. After removing excess DHE, cells were suspended in Phenol Red-free DMEM for 1 h prior to intensity measurement. Fluorescence microscopy for DHE was performed using a Nikon TE-2000 U fluorescence microscope with a 40× magnification 0.95-numerical-aperture objective lens. Images were acquired using a cooled CCD (charge-coupled-device) camera controlled by a computer that ran MetaVue imaging software (Universal Imaging). The fluorescence excitation source was controlled with a Uni-Blitz mechanical shutter. For image acquisition, a short exposure time (100 ms) and low-intensity excitation light were applied to minimize photo-bleaching. The fluorescent intensity of DHE in each cell was quantified by integrating the pixel intensity of the cell. Background subtraction was performed for each image prior to the quantification of the DHE intensity of cells. For each treatment group, we analyzed an average of ten images.

Cell adhesion

Cells were seeded into a 24-well plate (Falcon, Corning, US). The final cell density was 50,000 cells/well. Cells were incubated at 37°C, 5% CO_2, and 95% relative humidity for 5 h with the treatment of Mg^{2+}. Then, the cell medium was removed, and cells were washed by DPBS three times. Images of adhered cells were taken by a microscope (EVOS FL Cell Imaging System, AMG, US). The plate was sealed by self-sticking tape (Fisherbrand, Fisher Scientific, US). Then, the plate was put into the rotor inversely and centrifuged at 500 rpm for 5 min. Cells were washed by DPBS and fixed by 4% paraformaldehyde (Boston BioProducts, US). The images of adhered cells were taken by a microscope (EVOS FL Cell Imaging System, AMG, US) and analyzed by ImageJ (NIH, US). At least 10 different fields were used for calculating adhered cell density and cell retention ratio.[1-2]

Cell migration

Cells were seeded in a 12-well cell culture plate (BD Biosciences, US). A straight line in a cell monolayer was created by scratching the surface using a p200 pipette tip (Thermo Scientific, US). Debris was removed by gently washing with Dulbecco's Phosphate Buffered Saline (DPBS, Invitrogen, US) 3 times and cells were incubated with 3 ml medium supplemented with different ion solutions. At 0 and 6 hours, optical images were taken using a phase contrast microscope (Advanced Microscopy, US). The width of the line at upper, middle and bottom positions was measured in Image-Pro Plus 6.0 (Media Cybernetics, US). The average cell migration rate was calculated as described before.[1-2]

Real Time-PCR

Cells were seeded in 100 mm culture dishes (BD Technologies, US) and allowed to attach for 24 h. After different treatment, cells were harvested and total RNA was extracted by using a RNeasy Mini Kit (Qiagen, US) and subsequently quantified using a spectrophotometer (Nanodrop 2000, US) with OD260/OD280 ratios between 1.9 and 2.1. A total of 600 ng RNA was used for reverse transcription using a RT2 First Strand Kit (Qiagen, US). Reverse-transcription was performed in a thermo cycler (T100, Bio-Rad, US). Then 91 μl RNase-free water was added to the 20 μl cDNA mix and stored at -20°C in a freezer (Puffer Bubbard, Thermo Scientific, US). Real time PCR was used to estimate the mRNA levels of different gene expression in a CFX96 Touch RT-PCR Detection System (Bio-Rad, US). Total 25 μl PCR components mix including cDNA, SYBR Green Mastermix and RNase-free water was dispensed to PCR plate. After initial heat activation (95°C, 10 min), cDNA was amplified as the following parameters: 95°C for 15 s and 60°C for 1 min. After the amplification, melting curve analysis was performed using the default melting curve program. Data was analyzed by Bio-Rad CFX Manager 3.1 (Biorad, US). The $2{-}\Delta\Delta Ct$ method was used to calculate gene fold changes. The level of specific mRNA was normalized to the endogenous control, β-actin. Primers and probes were from Dharmacon RNAi Technologies (Thermo Scientific).[1-2]

Gene silencing

Cells were seeded in 60-mm cell-culture dishes (density of 7×10^5 cells/plate) or in 96-well plates (1.5×10^4 cells/well) 24 h before transfection and maintained in standard culture media as described above, without penicillin or streptomycin. Cells were transfected using Lipofectamine 2000 (Invitrogen, CA, US) according to the manufacturer's protocol, and used 48 h post

transfection. Small interfering RNA (siRNA) targeting human TRPM7, MagT1 and S1P1 were purchased from Santa Cruz Biotechnology. The corresponding scrambled siRNAs were used as controls.[4]

Measurement of [Mg^{2+}]$_i$ and [Ca^{2+}]$_i$

The selective fluorescent probes, mag fura-2AM and fura-2AM, were used to measure [Mg^{2+}]$_i$ and [Ca^{2+}]$_i$, respectively, as described.[5] [Mg^{2+}]$_i$ responses to increasing concentrations of extracellular Mg^{2+} (10 mmol/L) were measured in cells incubated in Mg^{2+}-free, Ca^{2+}-containing modified Hanks' buffer. Cells were exposed to Mg^{2+}-free buffer for 15 to 20 minutes before addition of extracellular Mg^{2+}. [Ca^{2+}]$_i$ responses to ionomycin (10^{-6} mol/L) were determined in cells incubated for 15 to 20 minutes in modified Hank's buffer containing Mg^{2+}(mmol/L, $MgCl_2$ 0.5 and $MgSO_4$ 0.8).

Transmonolayer electrical resistance (TER)

The cells were cultured on inserts with collagen-coated polycarbonate in Transwell. The transwell has membrane filters with 0.4 µm pores (Corning Life Science, Lowell, MA). TER was measured using an Endohmeter (World Precision Instruments, Sarasota, FL). TER values were expressed in $\Omega \cdot cm^2$. The resistance of collagen-coated inserts was subtracted from the resistance obtained in the presence of the endothelial cultures.[4]

Permeability of the endothelial barrier

We used fluorescein-isothiocyanate (FITC)-labeled dextran (40 kDa, Invitrogen) to measure permeability.[4] Briefly, the medium in upper chamber of an established monolayer was replaced

with 500 µL FITC-labeled dextran solution (2 mg/mL) in phenol-red free DMEM. The lower chamber containing 1,500 µL phenol-red free DMEM was sampled (20 µL) at 5 min intervals for 30 min. At each time point, 20 µL samples were taken from the upper chamber as well. The fluorescence intensity of each sample was determined using a fluorescence multiwell plate reader (Perkin Elmer Victor 3) at excitation/emission wavelength of 490/525 nm. The corresponding FITC-labeled dextran concentrations were determined from the standard curve. The endothelial monolayer permeability to dextran was expressed as a permeability coefficient in $cm \cdot s^{-1}$. The volume cleared (ΔV_c) of each time point was calculated using equation 1:

$$\Delta V_c = C_{lower} \times V_{lower} / C_{upper} \quad \text{(Eq. 1)}$$

where C_{upper} and C_{lower} are FITC-labeled dextran concentrations in upper and lower chambers respectively, and V_{lower} is the volume in lower chamber.

The volume cleared (ΔV_c) was plotted against time, and the permeability surface area (PS) product was obtained from the slope by linear regression. The permeability coefficient (P) was then calculated by equation 2:

$$P = PS/s \quad \text{(Eq. 2)}$$

where s is the surface area of the filter (1.12 cm^2).

Finally, the permeability coefficient of cells (P_{cell}) was obtained by correcting the overall permeability coefficient ($P_{cell+filter}$) for that of the cell-free filter (P_{filter}) using equation 3:

$$1/P_{cell} = 1/P_{cell+filter} - 1/P_{filter} \quad \text{(Eq. 3)}$$

P_{filter} was determined on a separate series of experiments using the cell-free filter inserts only.

Immunofluorescence and cytoskeleton staining

Cells were seeded in a 12-well cell culture plate and treated with medium supplemented with different treatments for 24 hours. An Image-iT Fix-Perm kit (Invitrogen, US) was used to fix cells. F-actin was stained by Actin Green 488 Ready Probes Reagent (Invitrogen, US). The cell nucleus was stained by the SlowFade Gold Anti-fade Reagent with DAPI (Invitrogen, US). The protein of interest was stained by primary antibody (Abcam, US) followed by Alexa Fluor 488 or 594 secondary antibody (Invitrogen, US). Images were taken using an EVOS Inverted Fluorescent Microscope (Advanced Microscopy, US). Fluorescent intensity of the cells was extracted by using ImageJ 1.49 software (NIH, US). Contrast of the representative images was auto-adjusted using Image-Pro Plus 6.0.[1-2]

siRNA knockdown

Endothelial cells were seeded into 6-well plate with 10% FBS DMEM without antibiotics. Cells were incubated at 37℃ to reach the confluency of 90%. Transfection complex was prepared by mixing solution B into solution A, and incubating for 15min at RT. Solution A: 250µl opti-MEM with 6µl lipo2000, incubate 15min at RT; solution B: 250µl opti-MEM with 6µl siRNA(10µM). A total volume of 512µl transfection mixture was added into each well, cells were incubated at 37℃ for 24 hours. After that, cells were harvested for western blot. Nucleotide sequence of siRNA is listed in **Table S1**.

Western Blot

Cells were lysed using cell lysis buffer (cell signaling technology) following the manufacturer’s protocol. After ultrasonication and centrifugation, proteins were extracted, and concentrations were measured using Micro BCA Protein Assay Kit (Thermo Scientific). After denaturation,

proteins were loaded to the 10% SDS-PAGE. After running the gel, proteins were transferred to the PVDF membrane and staining with selected antibody. Chemiluminescence images were acquired under darkroom development techniques (BIO-RAD).

cAMP direct immunoassay

The concentration of cAMP was measured following the instruction of cAMP Direct Immunoassay Kit (Abcam, US). Briefly, the cultured cells were scraped and dissociated completely, centrifuged at 14,000×g for 10 min to collect the supernatant as the testing sample. After neutralized and acetylated with Acetylating Reagent and Neutralizing Buffer, respectively, 50 μL of standard cAMP (or testing samples was added to the Protein G coated 96-well plate and incubated with 10 μL of cAMP antibody at room temperature for 1 h with gentle agitation. Then 10 μL of cAMP-HRP was added, and the plates were incubated for another hour. The suspension was discarded and the cells in the wells were washed with 1×cAMP Assay Buffer for five times. The detecting reaction was conducted by incubating the cells with 100 μL of HRP for 1 h and stopped by adding 100 μL of 1 M HCl. Then the reaction was checked by the microtiter plate reader at 450 nm. The absorbance of the substrate was also detected as background absorbance and subtracted from all standards and samples. The molar concentration of cAMP in cell pellets was determined from standard curves.[6]

Rac1 activation assay

The cells were washed using ice cold PBS and lysed with a lysis buffer supplied in the kit. Cell lysates were centrifuged at 12,000 g at 4°C for 5 min and supernatants incubated with PAK (p21 activated kinase 1 protein)-PBD [Rac/Cdc42 (p21) binding domain] beads at 4°C for 2 h. The

beads were washed three times. Rac bound to beads was extracted by boiling each sample in Laemmli sample buffer. Samples from beads and total cell lysates were electrophoresed on 15% SDS-PAGE gels, transferred to nitrocellulose, blocked with 5% nonfat milk and analyzed by Western blotting using a monoclonal anti-Rac1 antibody (supplied with the kit). In addition, cell lysates from samples were immunoblotted with anti-Rac antibody as a protein loading control in each lane.[4]

S1P1 threonine phosphorylation

Cells were lysed using RIPA buffer after specified treatments. Samples were immunoprecipitated using a Protein G immunoprecipitation kit (Roche Applied Sciences, Indianapolis, IN) with a rabbit polyclonal anti-human S1P1 antibody (Abcam, Cambridge, MA) followed by SDS-PAGE separation and transfer onto nitrocellulose membranes (Millipore Corp) and incubation with either anti-S1P1 antibody (Abcam, Cambridge, MA) or anti-phosphothreonine antibody (Cell Signaling, Danvers, MA). Following incubation with the corresponding secondary HRP-conjugated antibodies, the immunoreactive products were detected with a chemiluminescent kit (Pierce, Rockford, IL).[4]

Akt serine phosphorylation

Cells were lysed using RIPA buffer after specified treatment. Proteins (20 µg) were analyzed by 4-15% Tris-HCL gel and transferred to nitrocellulose membranes (0.45 µm, Bio-Rad Laboratories, Hercules, CA). The membranes were incubated overnight with primary rabbit polyclonal anti-mouse Phospho-Akt (Ser473) antibody or rabbit polyclonal anti-mouse Akt antibody diluted in 5% non-fat milk or 5% BSA in TBS, and then incubated with the

corresponding secondary HRP-conjugated antibodies for 1 h. Immunoreactivity was detected using the ECL detection system (Amersham, Piscataway, NJ).[4]

In vivo blood vessel permeability measurement using Miles assay

Miles assay is based on the intravenous injection of Evans Blue in mouse animal model.[7] Evans Blue is a dye that binds albumin. The endothelium is impermeable to albumin under normal conditions, therefore Evans blue bound albumin remains restricted within blood vessels. Under pathologic conditions, the endothelium becomes permeable to small proteins such as albumin, which allows for extravasation of Evans Blue in tissues. Thus, a healthy endothelium prevents extravasation of the dye in the neighboring vascularized tissues while organs with increased permeability will show significantly increased blue coloration. The level of vascular permeability can be assessed by quantitative measurement of the dye. When ready for in vivo experiments, mice were put into a restraint device so that the animal is not freely mobile but its tail can be handled. Briefly, 200 μL 0.5% sterile solution of Evans blue in PBS was intravenously injected into the mouse lateral tail vein. After 30 min, mice were sacrificed through cervical dislocation. For Miles assay purposes cervical dislocation is recommended as it limits significant interference with vascular permeability. Organs of interest were then collected, air dried, and incubated in formamide at 55ºC for 48 hr to extract Evans blue from tissues. After centrifugation, solutions were subjected to absorbance measurement at 610 nm. The amount of Evans Blue (ng) extravasated per mg tissue was then calculated.

Transient vessel permeability increase and Mg treatment in vivo

To evaluate the rescue effect of Mg on endothelial permeability in vivo, a transient leaky vessel model in mice were created by histamine treatment with or without Mg.[8] Mice were first pre-treated with Mg or saline control. $MgSO_4$ injection was administrated by an I.P. dose of 350 mg/kg followed by 50 mg/kg every 20 minutes for 4 hours; injections are given in a volume of 0.1 ml. A second dose of 350 mg/kg was given at the end of the 4 hour period. Mice were then injected with Evans Blue solution. To induce vascular hyper-permeability, 5 mg histamine (Sigma-Aldrich) dissolved in 500 μL PBS was injected via tail vein. After 30 min, mice were sacrificed and subjected to Miles assay. The S1P1 specific inhibitor W146 on Mg-mediated endothelial integrity was also assessed by pre-treating mouse with W146 (20 mg/kg) together with $MgSO_4$.

Table S1. Nucleotide sequence of siRNA

Nucleotide sequence of LTRPC7 siRNA targeting TRPM7 gene

siRNA	Nucleotide sequence
sc-42662A	5'-GAGAUGUGGUUGCUCCUUAtt-3' (sense)
	5'-UAAGGAGCAACCACAUCUCtt-3' (anti-sense)
sc-42662B	5'-CCAUAUUGGGUCAGAUGAAtt-3' (sense)
	5'-UUCAUCUGACCCAAUAUGGtt-3' (anti-sense)
sc-42662C	5'-GCAUUAGUUGCCUGUAAGAtt-3' (sense)
	5'-UCUUACAGGCAACUAAUGCtt-3' (anti-sense)

Note: LTRPC7 siRNA (h) is a pool of 3 different siRNA duplexes

Nucleotide sequence of MagT1 siRNA targeting MagT1 gene

siRNA	Nucleotide sequence
sc-91352A	5'-GAGAUGGUGUUAUCUGAAAtt-3' (sense)
	5'-UUUCAGAUAACACCAUCUCtt-3' (anti-sense)
sc-91352B	5'-CCGAGAAAUUACUCCGUUAtt-3' (sense)
	5'-UAACGGAGUAAUUUCUCGGtt-3' (anti-sense)
sc-91352C	5'-GCUUCUCGAUAGCUGUGAAtt-3' (sense)
	5'-UUCACAGCUAUCGAGAAGCtt-3' (anti-sense)

Note: MagT1 siRNA (h) is a pool of 3 different siRNA duplexes

Nucleotide sequence of SLC41A1 siRNA targeting SLC41A1 gene

siRNA	Nucleotide sequence
sc-88707A	5'-GACUCUACCUGGAACUGAAtt-3' (sense)
	5'-UUCAGUUCCAGGUAGAGUCtt-3' (anti-sense)
sc-88707B	5'-CUCUGCACUUUCUAUUUGAtt-3' (sense)
	5'-UCAAAUAGAAAGUGCAGAGtt-3' (anti-sense)
sc-88707C	5'-CCAAGUUUGUAUAGCAAGAtt-3' (sense)
	5'-UCUUGCUAUACAAACUUGGtt-3' (anti-sense)

Note: SLC41A1 siRNA (h) is a pool of 3 different siRNA duplexes

Nucleotide sequence of control siRNA

siRNA	Nucleotide sequence
	5'-UUCUCCGAACGUGUCACGUtt-3' (sense)
	5'-ACGUGACACGUUCGGAGAAtt-3' (anti-sense)

References

[1] a) J. Ma, N. Zhao, D. Zhu, *Journal of biomedical materials research. Part A* **2016**, 104, 347; b) N. Zhao, D. Zhu, *Metallomics : integrated biometal science* **2015**, 7, 118.

[2] a) J. Ma, N. Zhao, D. Zhu, *ACS Biomater Sci Eng* **2015**, 1, 1174; b) J. Ma, N. Zhao, D. Zhu, *Sci Rep* **2016**, 6, 26661; c) N. Zhao, N. Watson, Z. Xu, Y. Chen, J. Waterman, J. Sankar, D. Zhu, *PloS one* **2014**, 9, e98674; d) N. Zhao, B. Workman, D. Zhu, *International journal of molecular sciences* **2014**, 15, 5263; e) N. Zhao, D. Zhu, *PloS one* **2014**, 9, e110420.

[3] D. Zhu, C. Hu, W. Sheng, K. S. Tan, M. A. Haidekker, A. Y. Sun, G. Y. Sun, J. C. Lee, *The Biochemical journal* **2009**, 421, 201.

[4] D. Zhu, Y. Wang, I. Singh, R. D. Bell, R. Deane, Z. Zhong, A. Sagare, E. A. Winkler, B. V. Zlokovic, *Blood* **2010**, 115, 4963.

[5] Y. He, G. Yao, C. Savoia, R. M. Touyz, *Circulation research* **2005**, 96, 207.

[6] D. Zhu, Y. Su, M. L. Young, J. Ma, Y. Zheng, L. Tang, *ACS Appl Mater Interfaces* **2017**, 9, 27453.

[7] M. Radu, J. Chernoff, *Journal of visualized experiments : JoVE* **2013**, DOI: 10.3791/50062e50062.

[8] a) C. Lu, S. A. Diehl, R. Noubade, J. Ledoux, M. T. Nelson, K. Spach, J. F. Zachary, E. P. Blankenhorn, C. Teuscher, *Proceedings of the National Academy of Sciences of the United States of America* **2010**, 107, 18967; b) G. P. van Nieuw Amerongen, R. Draijer, M. A. Vermeer, V. W. van Hinsbergh, *Circulation research* **1998**, 83, 1115.
